# Adenosine stress native T1 mapping in severe aortic stenosis: evidence for a role of the intravascular compartment on myocardial T1 values

**DOI:** 10.1186/s12968-014-0092-y

**Published:** 2014-11-20

**Authors:** Masliza Mahmod, Stefan K Piechnik, Eylem Levelt, Vanessa M Ferreira, Jane M Francis, Andrew Lewis, Nikhil Pal, Sairia Dass, Houman Ashrafian, Stefan Neubauer, Theodoros D Karamitsos

**Affiliations:** University of Oxford Centre for Clinical Magnetic Resonance Research (OCMR), Division of Cardiovascular Medicine, Radcliffe Department of Medicine, John Radcliffe Hospital, Oxford, OX3 9DU UK; 1st Department of Cardiology, AHEPA Hospital, Aristotle University of Thessaloniki, Thessaloniki, 54636 Greece

**Keywords:** Adenosine vasodilator, Intravascular compartment, Myocardial T1, Perfusion reserve, T1 mapping

## Abstract

**Background:**

Myocardial T1 relaxation times have been reported to be markedly abnormal in diverse myocardial pathologies, ascribed to interstitial changes, evaluated by T1 mapping and calculation of extracellular volume (ECV). T1 mapping is sensitive to myocardial water content of both intra- and extracellular in origin, but the effect of intravascular compartment changes on T1 has been largely neglected. We aimed to assess the role of intravascular compartment on native (pre-contrast) T1 values by studying the effect of adenosine-induced vasodilatation in patients with severe aortic stenosis (AS) before and after aortic valve replacement (AVR).

**Methods:**

42 subjects (26 patients with severe AS without obstructive coronary artery disease and 16 controls) underwent cardiovascular magnetic resonance at 3 T for native T1-mapping (ShMOLLI), first-pass perfusion (myocardial perfusion reserve index-MPRI) at rest and during adenosine stress, and late gadolinium enhancement (LGE).

**Results:**

AS patients had increased resting myocardial T1 (1196 ± 47 ms vs. 1168 ± 27 ms, p = 0.037), reduced MPRI (0.92 ± 0.31 vs. 1.74 ± 0.32, p < 0.001), and increased left ventricular mass index (LVMI) and LGE volume compared to controls. During adenosine stress, T1 in AS was similar to controls (1240 ± 51 ms vs. 1238 ± 54 ms, p = 0.88), possibly reflecting a similar level of maximal coronary vasodilatation in both groups. Conversely, the T1 response to stress was blunted in AS (ΔT1 3.7 ± 2.7% vs. 6.0 ± 4.2% in controls, p = 0.013). Seven months after AVR (n = 16) myocardial T1 and response to adenosine stress recovered towards normal. Native T1 values correlated with reduced MPRI, aortic valve area, and increased LVMI.

**Conclusions:**

Our study suggests that native myocardial T1 values are not only influenced by interstitial and intracellular water changes, but also by changes in the intravascular compartment. Performing T1 mapping during or soon after vasodilator stress may affect ECV measurements given that hyperemia alone appears to substantially alter T1 values.

## Background

Myocardial T1 determination using cardiovascular magnetic resonance (CMR) is a technique that can be used to provide a quantitative measure of myocardial water, without the need for exogenous contrast agents [1]. Native (pre-contrast) T1 values are elevated in conditions associated with increased myocardial water content such as acute myocardial infarction [2,3], oedema [4], acute myocarditis [5] and Takotsubo cardiomyopathy [6]. T1 values have also been shown to be elevated in conditions associated with myocardial fibrosis and expanded interstitial space such as dilated cardiomyopathy, hypertrophic cardiomyopathy [7] and aortic stenosis [8]. Other conditions associated with T1 changes are protein deposition in amyloid (increased T1 values) [9], and lipid deposition in Anderson-Fabry disease [10] and iron deposition due to hemorrhage (low T1 values) [11].

Despite the rapidly growing applications, the T1 sensitivity to detect disease is not fully understood and the measurements depend somewhat on the particular choice of methods [12–14]. In particular, the documented native T1 elevation in diffuse fibrosis cannot originate directly from collagen itself, which in large enough concentrations would tend to reduce T1 similarly to other proteins [15]. Thus, the observed increase in native T1 values may be due to increase in tissue water content independently of its extra/intracellular or intra/extravascular origin. However, the influence of intravascular water volume to the T1 values has never been investigated before, either in normal or pathological conditions.

In this work, we aimed to examine the impact of induced vasodilatation on the native T1 values following adenosine stress perfusion. This allowed assessment of the potentially reduced vascular reactivity in AS due to coronary microvascular dysfunction [16]. We also investigated if these changes were reversible after aortic valve replacement (AVR). We hypothesized that T1 values depend on the degree of coronary vascular recruitment and would increase under adenosine stress perfusion, and that the response would be blunted in AS when compared to normal.

## Methods

### Study population

We prospectively recruited 26 patients with severe AS without obstructive coronary artery disease (CAD) on invasive coronary angiography scheduled prior to AVR. Severe AS was diagnosed based on the previously established criteria [17]. Briefly patients were included if the aortic valve area (AVA) was ≤1.0 cm^2^, peak aortic valve gradient was 64 mmHg and there was no other significant valvular pathology on echocardiogram. Patients were excluded if they had systolic blood pressure (BP) >160 mmHg and diastolic BP >90 mmHg, LVEF <50%, contraindications to CMR, glomerular filtration rate <60 ml/min, underlying cardiomyopathy or known history of CAD. Of these patients, 16 had a repeat CMR scan 7 months after AVR. Ten patients did not have a follow-up scan due to reasons such as death (2 patients), pacemaker implantation (1 patient), hospital admission due to unrelated to cardiac causes (1 patient), lost to follow-up (3 patients) and declined a repeat scan (3 patients). In addition, healthy volunteers with no prior cardiac history or known cardiac risk factors, not on any cardiovascular medications and with a normal ECG served as controls. All subjects gave written informed consent to participate in the study and ethical approval from the National Research Ethics Service committee South Central – Berkshire was granted for all study procedures.

### Cardiovascular magnetic resonance

CMR was performed in a 3 Tesla MR system (TIM Trio; Siemens Healthcare, Erlangen, Germany). Study participants were instructed to avoid caffeine-containing food and drinks for at least 24 hours prior to CMR scan. Cine imaging was performed using standard methods [18]. At rest, 3 short-axis images of T1 maps were acquired using the shortened modified look-locker inversion recovery (ShMOLLI) sequence as previously described [19]. Adenosine stress perfusion imaging was performed as previously described [20,21]. Briefly, adenosine (140 μg/kg/min) was infused intravenously for at least 3 minutes, followed by acquisition of a single mid-ventricular slice (identical to the one acquired at rest) of T1 map. Then stress perfusion imaging was performed immediately after stress T1 imaging, using a bolus a gadolinium-based contrast (Gadodiamide, Omniscan; GEHealthcare) at a dose of 0.03 mmol/kg followed by a saline flush. After discontinuing adenosine, rest perfusion images were acquired 20 minutes after the stress study to allow sufficient time for contrast washout. For late gadolinium enhancement (LGE), an additional bolus of 0.1 mmol/kg gadodiamide was administered (total dose: 0.16 mmol/kg) immediately after rest perfusion. The LGE images were acquired as previously described [22].

### Cardiac magnetic resonance image analysis

Analysis of cardiac volumes, function and mass was performed according to the standard methods [18]. AVA was measured from direct planimetry in cine imaging. Information on the peak aortic valve gradient was obtained from the clinical echocardiogram performed as part of routine clinical care. For the analysis of ShMOLLI T1-maps, the LV myocardium of the mid-axial slice acquired at baseline was contoured by a blinded observer (EL) using dedicated software, as previously described [6] providing a single average myocardial T1 value per each individual. The average values were also calculated after manually excluding areas with focal LGE. The myocardial T1 value from the stress mid-ventricular ShMOLLI T1 map was obtained and compared to the T1 at baseline. For perfusion analysis, briefly, 3 contours (epicardial, endocardial and LV blood pool) were drawn on a single image and propagated throughout the perfusion series. The myocardium was divided into segments based on the American Heart Association (AHA) segmentation model. Signal intensity (SI) versus time curves were generated and normalized to the left ventricular (LV) blood pool upslope. Myocardial perfusion reserve index (MPRI) was defined as the ratio of stress to rest relative upslope, as previously described [23]. LGE quantification was performed using cmr42 software (version 4.0, Circle Cardiovascular Imaging) by a blinded observer (AL). The full-width-half-maximum (FWHM) technique was used to quantify fibrosis as previously described [24]. The volume of focal fibrosis was expressed as the LGE mass (g) and also as a percentage of total myocardial mass.

### Statistical analysis

All data are expressed as mean ± standard deviation or median (interquartile range), and checked for normality using Kolmogorov–Smirnov test. Categorical data are presented as numbers and percentages. Comparisons between the 2 groups were performed using Student *t*-test, the chi-square test, or Fisher’s exact test as appropriate. Bivariate correlations were performed using Pearson’s or Spearman’s method as appropriate. Comparisons between pre- and post-AVR measurements in AS patients and rest and stress T1 values were performed using 2-tailed paired *t*-test. Comparisons between AS patients and controls at baseline and post-AVR were performed using Student *t*-test or Mann–Whitney U test as appropriate. A *P*-value <0.05 was considered significant. All statistical analyses were performed with IBM SPSS Statistics, version 20.

## Results

### Baseline study characteristics

Patient characteristics during the baseline examination are summarized in Table 1. There were no significant differences in age, sex, body mass index, BP and pulse rate from controls. In the AS group 38% were hypertensive but their BP were well controlled. There were a small number of diabetics (15%) in the AS patient group, but their blood glucose and lipid levels were similar to the controls (results not shown).

**Table 1:** **Baseline clinical characteristics of severe AS patients and normal controls**

	**Severe aortic stenosis (n = 26)**	**Normal controls (n = 16)**	**P value**
Age (years)	67.8 ± 9	63.3 ± 3.4	0.06
Male, n (%)	19 (73)	8 (53)	0.16
Past medical history, n (%)			
Hypertension	10 (38)	−	
Diabetes	4 (15)	−	
Medications, n (%)			
Metformin	4 (15)	−	
ACE-I/ARB-II	7 (27)	−	
Beta-blockers	5 (19)	−	
Body mass index (kg/m^2^)	27.8 ± 4.5	27.0 ± 3.8	0.38
Systolic blood pressure (mmHg)	134.4 ± 18.1	131.0 ± 11.0	0.51
Diastolic blood pressure (mmHg)	74.4 ± 9.4	76.5 ± 10.2	0.51
Heart rate (bpm)	66.1 ± 9.4	64.3 ± 10.5	0.58
Peak AV gradient (mmHg)*	83.1 ± 14.6	−	
CMR findings			
Aortic valve area (cm^2^)	0.82 ± 0.02	4.04 ± 0.75	<0.001
LV end-diastolic volume (ml)	143.2 ± 44.4	133.7 ± 33.1	0.47
LV ejection fraction (%)	74.5 ± 5.8	68.8 ± 6.4	0.005
LV mass index (g/m^2^)	96.0 ± 31.2	55.8 ± 13.9	<0.001
Presence of LGE, n (%)	21 (81)	0	<0.001
Myocardial T1 (ms)	1196 ± 47	1168 ± 27	0.037

Values are mean ± SD or percentages. ACE indicates angiotensin-converting enzyme-inhibitors; ARB, angiotensin-receptor antagonist-II; LGE, CMR, cardiovascular magnetic resonance; late gadolinium enhancement; LV, left ventricular.

*Based on echocardiogram.

### Assessment of left ventricular mass, function, focal fibrosis and myocardial T1

CMR results are also summarized in Table 1. AS patients had significantly higher LVMI and LV ejection fraction when compared to controls. There was high burden of myocardial fibrosis in AS patients (absolute volume of focal fibrosis 29.3 g, IQR 16.8-58.8 g; % LV mass 18.3 ± 9.4%). None of the controls had LGE. As previously reported [8], native myocardial T1 was significantly higher in AS patients when compared to normal controls and remained elevated after excluding the areas with focal LGE (1199 ± 46 ms, p = 0.025 vs. normal controls).

### Myocardial T1 and perfusion reserve under adenosine vasodilator stress

Both AS patients and controls had equivalent rises in rate pressure product (RPP) during adenosine stress (baseline 8800 ± 1600 mmHg · beats/min in AS vs. 8100 ± 1500 mmHg · beats/min in controls, p = 0.19) and (stress 11,400 ± 2500 mmHg · beats/min in AS vs. 11800 ± 2800 mmHg · beats/min in controls, p = 0.69).

Importantly we now report that T1 values were significantly increased during adenosine stress in both AS patients (from 1196 ± 47 ms to 1240 ± 51 ms, p < 0.001) and normal controls (from 1168 ± 27 ms to 1238 ± 54 ms, p < 0.001). Interestingly, both groups exhibited similar maximal T1 values during adenosine stress (1240 ± 55 ms in AS vs. 1238 ± 54 ms in normal controls, p = 0.90). Given the T1 increase at rest, the stress response was blunted in AS patients, as expressed by significantly smaller absolute ΔT1 (44 ± 33 ms in AS vs. 70 ± 49 ms in normal controls, p = 0.013) and percentage of ΔT1 (3.7 ± 2.7% in AS vs. 6.0 ± 4.2% in normal controls, p = 0.013). As expected, myocardial perfusion reserve was significantly reduced in AS patients when compared to normal controls (MPRI 0.92 ± 0.31 vs. 1.74 ± 0.32, p < 0.001).

### Relationship amongst T1 values, perfusion and focal fibrosis (LGE)

#### *Resting T1*

Resting T1 values increased with increasing LVMI (*r* = 0.59, p < 0.001) and absolute LGE volume (*r* = 0.46, p = 0.03) but not LGE volume as % of LV mass (r = 0.41, p = 0.066). Furthermore, T1 values had an inverse correlation with MPRI (*r* = −0.51, p = 0.001) and AVA (−0.35, p = 0.026). These relationships and their significance were preserved when the correlation analysis was repeated excluding segments showing focal LGE (results not shown).

#### *Stress T1*

Stress T1 values had no significant correlations with LVMI, MPRI or LGE. The degree of stress T1 response (%ΔT1) showed weak negative correlation with LVMI but no significant correlation with MPRI or LGE.

### Cardiac MRI post AVR (n = 16)

**Figure 1:**
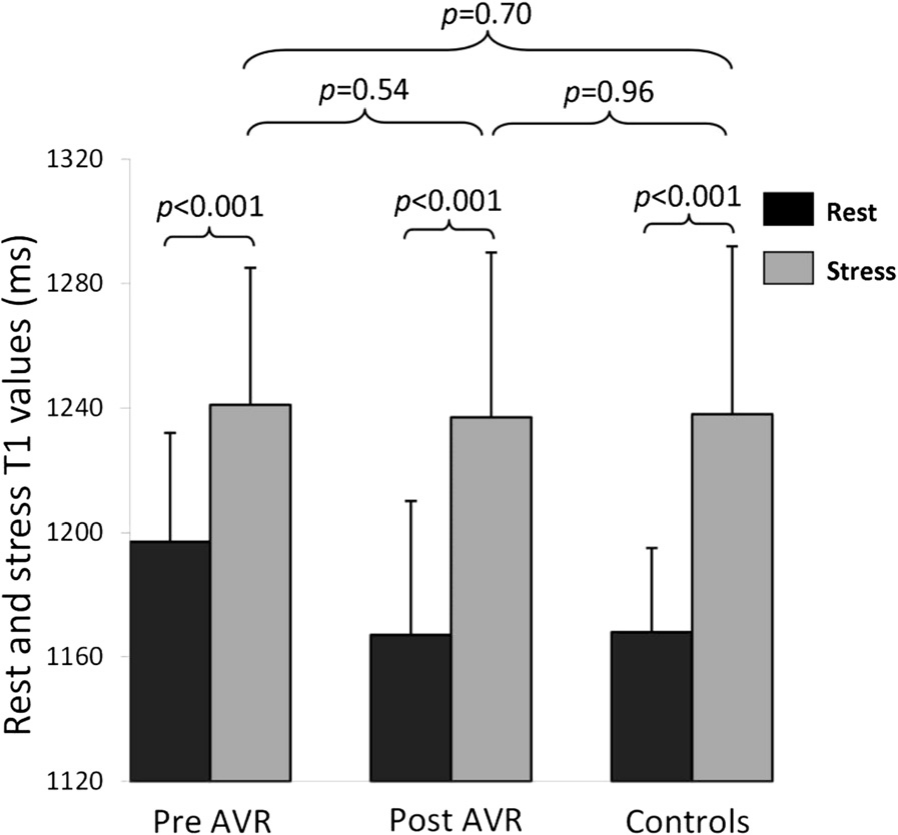
**Rest and adenosine stress T1 values.** Changes in myocardial T1 values (rest and stress) before and after aortic valve replacement and normal controls. Error bars represent the 95% confidence interval for the mean.

**Figure 2:**
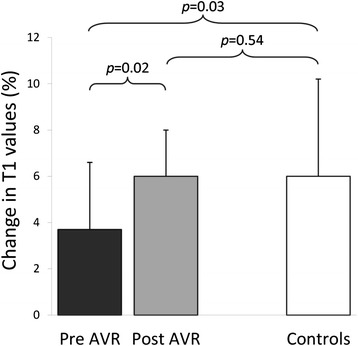
**Myocardial T1 reactivity to adenosine stress pre and post AVR.** Error bars represent the 95% confidence interval for the mean.

**Figure 3:**
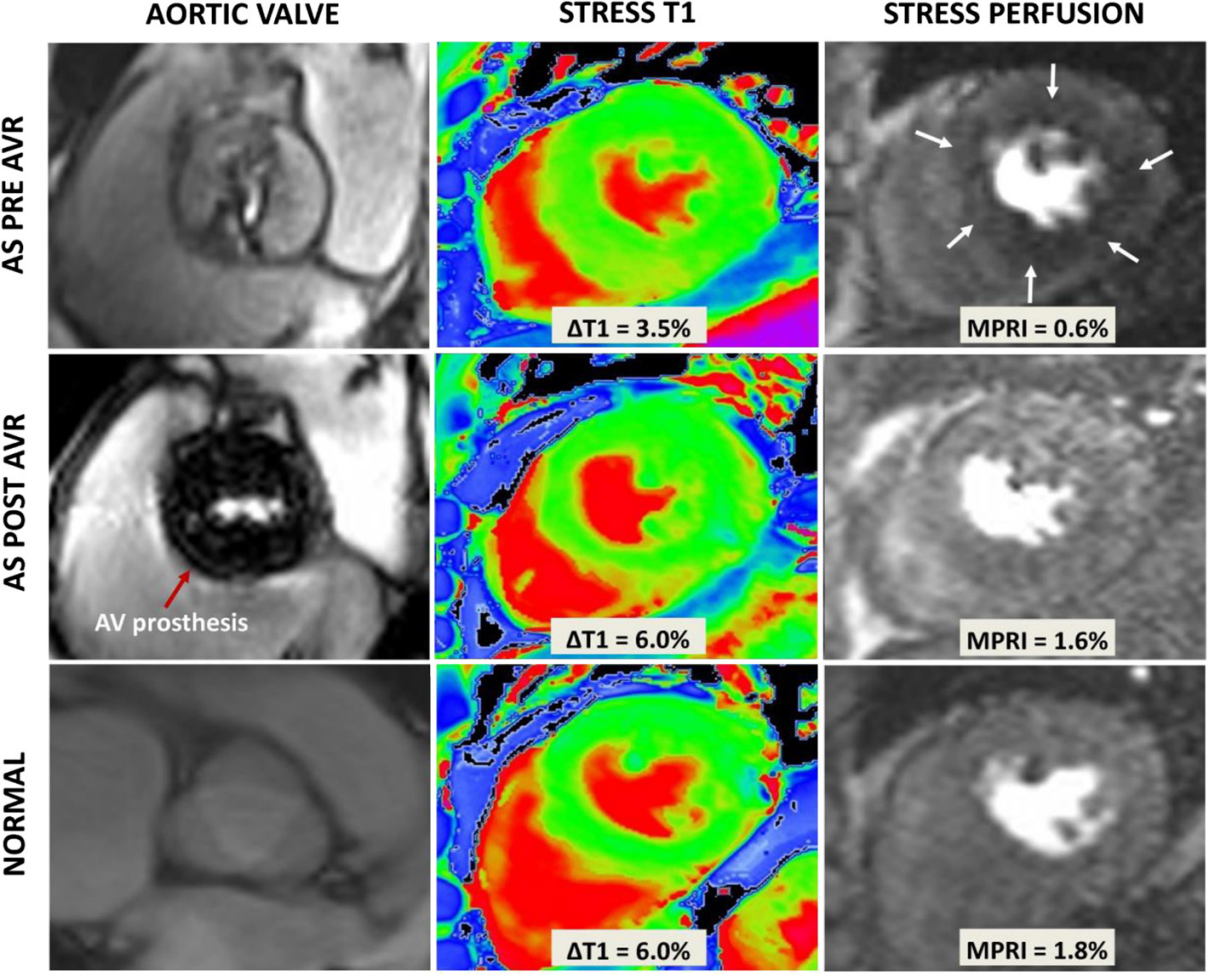
**Examples of stress T1 imaging.** Cardiovascular magnetic resonance images show examples of AS pre and post AVR, and normal (from top to bottom panels). From left to right, examples of AV cine, stress T1 (with ∆T1 values between rest and stress), and stress perfusion imaging (with MPRI values). White arrows show circumferential perfusion defect in AS pre AVR which is not seen in post AVR or normal. AS = aortic stenosis; AVR = aortic valve replacement; AV = aortic valve; ∆T1 = change in T1; MPRI = myocardial perfusion reserve index.

These results are summarized in Table 2. As expected, there was substantial reduction of LVH and improvement in perfusion reserve 7 months following AVR. There was significant reduction in resting T1 values post AVR with no significant difference compared to controls. More importantly, the diminished T1 response to adenosine stress pre-AVR improved significantly to normal level post-AVR (Figures 1 and 2), as shown by an increase in T1 reactivity (absolute ΔT1 44 ± 34 ms pre-AVR to 70 ± 24 ms post-AVR, p = 0.02 [paired t test for n = 16]; post-AVR ΔT1 vs. controls, p = 0.57). Examples of CMR images of a patient with severe AS pre and post AVR and a normal control are shown in Figure 3. The absolute volume of focal fibrosis decreased post-AVR, but the percentage LV myocardium that showed fibrosis was unchanged compared to pre-AVR.

**Table 2:** **CMR and before and after AVR (n = 16)**

	**Pre AVR**	**Post AVR**	**Normal**
Myocardial T1 (ms)	1197 ± 35	1167 ± 43*	1168 ± 27
Stress myocardial T1 (ms)	1241 ± 44	1237 ± 53^†^	1238 ± 54
∆T1 (%)	3.7 ± 2.9	6.0 ± 2.0*	6.0 ± 4.2
Myocardial perfusion reserve index	0.97 ± 0.38	1.63 ± 0.56^*^	1.74 ± 0.32
Left ventricular mass index (g/m^2^)	97.4 ± 29.0	68.8 ± 16.4^‡^	55.8 ± 13.9
Absolute LGE Mass (g)	25.0 (14.9-54.3)	17.3 (13.6-30.4)**	-
LGE % of myocardium (%)	17.5 ± 10.6	16.4 ± 10.8***	-

Values are mean ± SD or median (interquartile range). LGE indicates late gadolinium enhancement.

*p < 0.05 vs pre AVR and >0.05 vs normal; †p > 0.05 vs pre AVR and normal; ‡p < 0.05 vs pre AVR and normal; **p < 0.05 vs pre AVR; ***p > 0.05 vs pre AVR. LGE data were based on n = 14 who had LGE + .

## Discussion

Increased myocardial T1 values has previously been linked to expansion of interstitial space owing to interstitial fibrosis based on native T1 and ECV measurements using post-contrast T1 techniques [25,26]. In this study, we confirm that resting myocardial T1 values are elevated in severe AS patients compared to normal controls and correlate with markers of disease severity, such as aortic valve area, LVH [8], and myocardial perfusion reserve. We have shown for the first time that T1 values in both AS patients and normal controls increase in response to adenosine vasodilator stress. Interestingly, the achieved level of maximal stress T1 was the same in AS patients as in controls, with blunted stress T1 response (reduced ΔT1) in AS owing to higher resting T1. Importantly, the abnormal resting myocardial T1 and the reduced ΔT1 stress response normalized seven months post-AVR. We did not find a significant reduction in focal fibrosis (based on LGE% of LV mass) post AVR, in line with a previous study [27]. Furthermore, others showed no significant diffuse fibrosis regression 6 months post AVR measured by equilibrium contrast [28]. These findings are hypothesis-generating, raising the possibility that, in severe AS, increased resting myocardial T1 may have a signal origin specifically in the intravascular compartment, rather than be explained solely by diffuse myocardial fibrosis in the interstitial compartment which we did not expect previously [7,8] and also as suggested by others [8,26,28]. Increased resting coronary blood flow in severe AS as adaptation for the increased demands of the hypertrophied myocardium has previously been demonstrated [16,29–31], despite inadequate growth of vascular bed, characteristic of pressure overload hypertrophy [32].

### Influence of intravascular myocardial blood volume on myocardial T1 relaxation times

T1-mapping is typically known for edema imaging and as a surrogate for diffuse myocardial fibrosis in the absence of other causes of interstitial expansion (such as edema and amyloidosis) [1], but in principle it measures global water content in tissue, whether intra-cellular, extra-cellular or intravascular. To our knowledge, the influence of intravascular myocardial blood volume and its dynamic changes on myocardial T1 has never been investigated previously. In this work we used a vasodilator stress agent, adenosine [33] to study directly the vascular compartment contribution to the native T1 in both normal and diseased conditions. We demonstrate for the first time a clear effect of the intravascular compartment during stress on myocardial T1 in normal controls and AS patients. The attenuated stress T1 response in severe AS may reflect reduced myocardial perfusion reserve in line with previous studies [16,34]. This observation paves way for coronary reserve assessments with stress T1-mapping as an alternate biomarker, free of intravascular administration of Gadolinium contrast, and insensitive to potential heart rate dependencies due to properties of the underlying T1 mapping technique [19].

### Diffuse myocardial fibrosis may not be the sole contributor to increased myocardial T1 and extracellular volume in severe AS

The increased native T1 and ECV in AS is traditionally attributed to interstitial expansion due to diffuse fibrosis. However, if T1 changes were due to interstitial expansion and diffuse fibrosis only, then we would expect an increase in T1 values in AS both at baseline and during the stress due to presence of increased interstitial water. In contrast, we found that stress T1 values were similar for control and AS patients. The complete normalization of both resting T1 and vasodilator T1 response after AVR although the percentage of LV myocardium that shows focal fibrosis is unchanged indicates a significant role of the intravascular compartment in myocardial tissue pathology in AS. This concurs with a previous study by Eberli *et al.* who demonstrated markedly increased resting blood flow and reduced coronary flow reserve in aortic valve disease, but maximum flow during stress is similar to patients who had valve replacement and normal controls [30].

**Figure 4:**
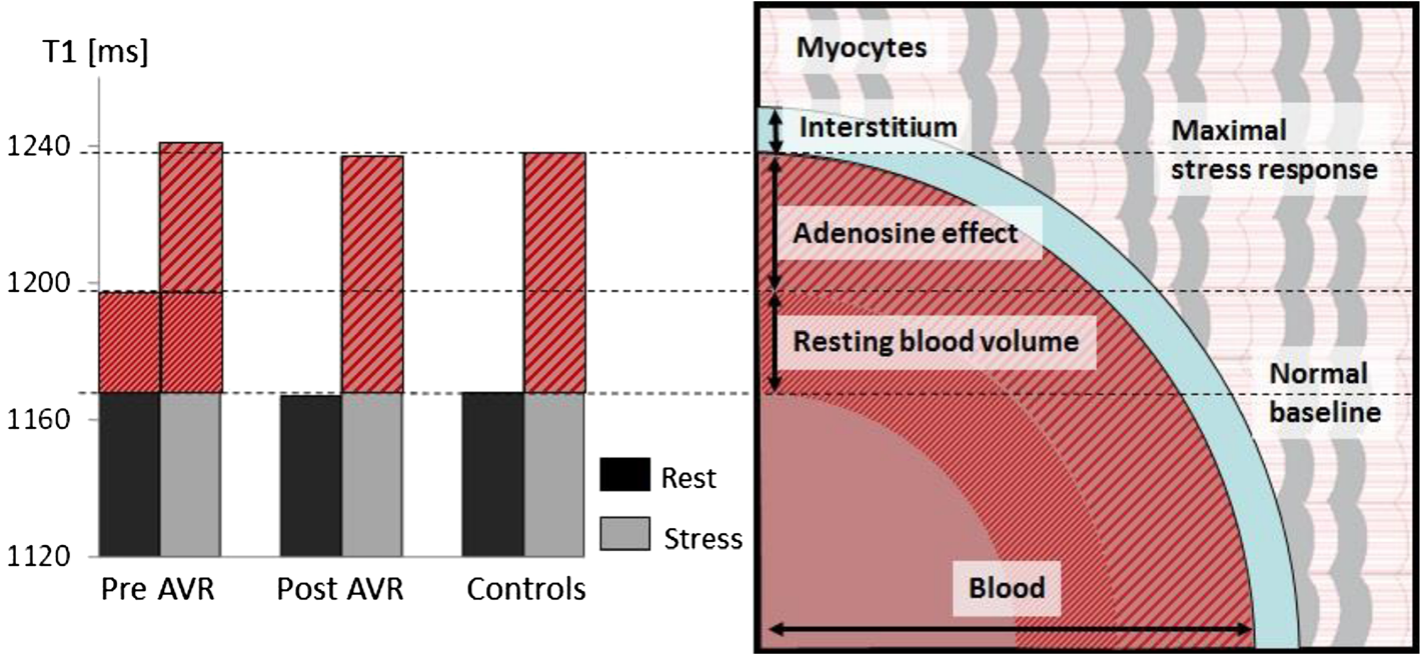
**Proposed myocardial water compartments in aortic stenosis.** Proposed changes in myocardial water compartments at rest and stress in patients with aortic stenosis pre and post AVR, and controls (left – adapted from Figure 1). The T1 response to adenosine was mainly contributed to by vascular responses instead of interstitial space expansion which may be negligible. Note that T1 and volumes from vascular cross-sections are for qualitative comparison only and not to scale.

Figure 4 illustrates the proposed changes of the water compartment in the myocardial tissue of AS patients. This scenario is fully consistent with the interpretation of the impact of the reactive partial blood volume of blood, which has significantly more water content and a longer T1 than the mix of tightly packed myocytes separated by the true interstitial space. The effective myocardial T1 resulting from such simplified two-compartmental model can be expressed as: [35,36].

$$ T{1}_{myocardium}\sim P{V}_{Blood}\cdotp T{1}_{Blood}+P{V}_{Cells\& Interstitium}\cdotp T{1}_{Cells\& Interstitium} $$

$$ \mathrm{T}{1}_{\mathrm{blood}}\sim 1800\mathrm{ms}\ \mathrm{at}\ 3\mathrm{T}\ \left(\mathrm{own}\ \mathrm{unpublished}\ \mathrm{data}\right)\ \mathrm{and}\ {\mathrm{PV}}_{\mathrm{blood}}\sim 10\%\ \mathrm{at}\ \mathrm{rest} $$

$$ \mathrm{T}{1}_{\mathrm{cells}\&\mathrm{interstitium}}\sim 1100\mathrm{ms}\ \left(\mathrm{t}\mathrm{o}\ \mathrm{fit}\ \mathrm{o}\mathrm{bserved}\ \mathrm{o}\mathrm{verall}\ \mathrm{T}1\ \mathrm{in}\ \mathrm{normal}\ \mathrm{controls}\ \mathrm{at}\ \mathrm{rest}\right) $$

The change in partial blood volume itself is hard to measure directly and is usually approximated as about a third of the corresponding change in the flow [37]. Assuming the maximal blood flow change in normal controls is about 3 fold [38], the normal resting blood volume in the myocardium should approximately double to 20% in normal controls with the respective simulated T1 reactivity ~6%, in excellent agreement with our observations. In AS patients before AVR, the same model implicates that the resting blood volume increased to ~14%, with respective flow response reduced to ~110%, in order to approximate the observed stress T1 reactivity at ~3.5%.

Our findings suggest that whenever adenosine stress is part of a CMR protocol that includes T1 mapping for ECV estimation, post contrast T1 should be acquired after a sufficient time has passed to ensure that myocardium is no longer in hyperemic state. Furthermore, our findings may also have important clinical implications as T1 changes in AS may serve as a marker for determining disease progress and therapeutic response, although this should be explored further in larger studies with a wider spectrum of AS severity.

### Study limitations

Our study population was small in line with a proof of concept study, and therefore confirmation in larger scale studies is warranted. We performed a single mid-ventricular short-axis T1-map during stress, which represents a sample of the myocardium rather than the entire left ventricle; however we believe that the myocardial changes in severe AS are mostly homogenous, and this is therefore unlikely to affect the results. Although good agreement of normal resting myocardial T1 has been documented between ShMOLLI and MOLLI T1-mapping techniques [19], the numeric results in T1 in disease and T1 vascular reactivity may not hold between different methods where multiple parametric dependencies between T1, T2 and magnetization transfer (MT) in multiple tissue compartments may affect the effect size [8,13,14,39,40]. Further research is required to fully model the interaction between flow, volume and blood T1, which have been shown to be strongly stratified by vessel size in the brain, but not deeply studied in the heart [37].

## Conclusions

Patients with severe aortic stenosis demonstrated increased resting myocardial T1 values, which correlated with aortic valve area, LVH and impaired myocardial perfusion reserve, but achieved the same maximal stress T1 response as healthy controls. The abnormal resting myocardial T1 and ΔT1 stress response normalized seven months post-AVR without specific treatment towards regression of myocardial fibrosis. Our findings support the concept that the intravascular compartment is a significant contributor to myocardial T1 relaxation times, and provide a novel mechanism to explain the T1 changes previously only attributed to diffuse myocardial fibrosis in the interstitial compartment. Coronary reserve may be assessed using native T1-mapping and this may serve as an alternate biomarker for disease severity and therapeutic response in aortic stenosis.

## Abbreviations

AS: Aortic stenosis
AVR: Aortic valve replacement
CMR: Cardiovascular magnetic resonance
ECV: Extracellular volume
FWHM: Full width half maximum
LGE: Late gadolinium enhancement
LVEF: Left ventricular ejection fraction
LVH: Left ventricular hypertrophy
LVMI: Left ventricular mass index
MPRI: Myocardial perfusion reserve index
ShMOLLI: Shortened modified look-locker inversion recovery
SI: Signal intensity
